# Early identification of postpartum depression using machine learning

**DOI:** 10.1111/pcn.13659

**Published:** 2024-04-15

**Authors:** Yukako Nakamura, Taro Ueno, Nagahide Takahashi, Daisuke Ichikawa, Aya Yamauchi, Norio Ozaki

**Affiliations:** ^1^ Department of Psychiatry Nagoya University Graduate School of Medicine Nagoya Japan; ^2^ SUSMED, Inc. Tokyo Japan; ^3^ Department of Child and Adolescent Psychiatry Nagoya University Graduate School of Medicine Nagoya Japan; ^4^ Psychiatry/Child and Adolescent Psychiatry Nagoya University Hospital Nagoya Japan; ^5^ Pathophysiology of Mental Disorders Nagoya University Graduate School of Medicine Nagoya Japan

During the perinatal period, the risk of developing depression is high and it is estimated that approximately 10%–15% of mothers experience perinatal depression.^1^ In recent years, machine learning has been widely used in the research of mental health, and it has been suggested that machine learning could be useful in the clinical management of mental disorders by providing accurate predictions for the diagnosis, prognosis. If an effective postpartum depression (PPD) prediction model can be established, it will enable early identification of high‐risk individuals and early intervention by healthcare providers in high‐risk individuals.^2^

In this study, we used machine learning methods to construct a prediction model for depression in the first postpartum month using demographic information and subjective ratings of pregnant women collected from the time of pregnancy to the fifth postpartum day after delivery. A verbal and written explanation of the study was given to all participants, and written informed consent was obtained from all those who agreed to participate. The study protocol was approved by the Ethics Committee of the Nagoya University Graduate School of Medicine. Detailed methods are described in the Supplementary materials Appendix S1. The flowchart of the study procedures is shown in Fig. S1. 1559 women participated in the study and 1416 women responded to all 10 Edinburgh Postnatal Depression Scale items 1 month after delivery. In this study, we included these 1416 women (mean age 32.4 years, standard deviation ±4.6 years) in our machine learning. The flowchart of the recruitment process is shown in Fig. S2. We show the method details in Appendix S1 and comparison of predictors between the PPD group and the non PPD group in Table S1. We also show missing percentages for each item in Table S3.

We used a machine learning approach, logistic regression, decision tree, gradient‐boosting decision tree (GBDT), and balanced GBDT to develop the PPD prediction model. GBDT gives a predictive model as an ensemble of decision tree and achieves high predictive ability with a differentiable loss function. Early prediction models for PPD need to be sensitive so as not to overlook women at high risk for PPD. Therefore, we set sensitivity to 80% and built the model using the machine learning approach. We calculated a 95% confidence interval for the AUC to confirm the predictive power of the model used. The results are shown in Table S2. The high accuracy (73.11%), high specificity (71.3%) and high positive predictive value (42.2%) was obtained in the balanced GBDT, and the high AUC value (0.8285) was obtained from the logistic regression model. Therefore, we thought that the balanced GBDT model would be the best. Figure 1 shows the feature value with high importance and AUC when using the balanced GBDT. In addition, we used the 28 features shown in Fig. 1 as predictors and built a predictive model again using the balanced GBDT. From the results, we were able to build a model with high accuracy and good sensitivity and specificity (accuracy 78.6%, AUC 0.83, sensitivity 71.1%, specificity 80.6%, positive predictive value 48.9%, negative predictive value 91.4%).

**Fig. 1:**
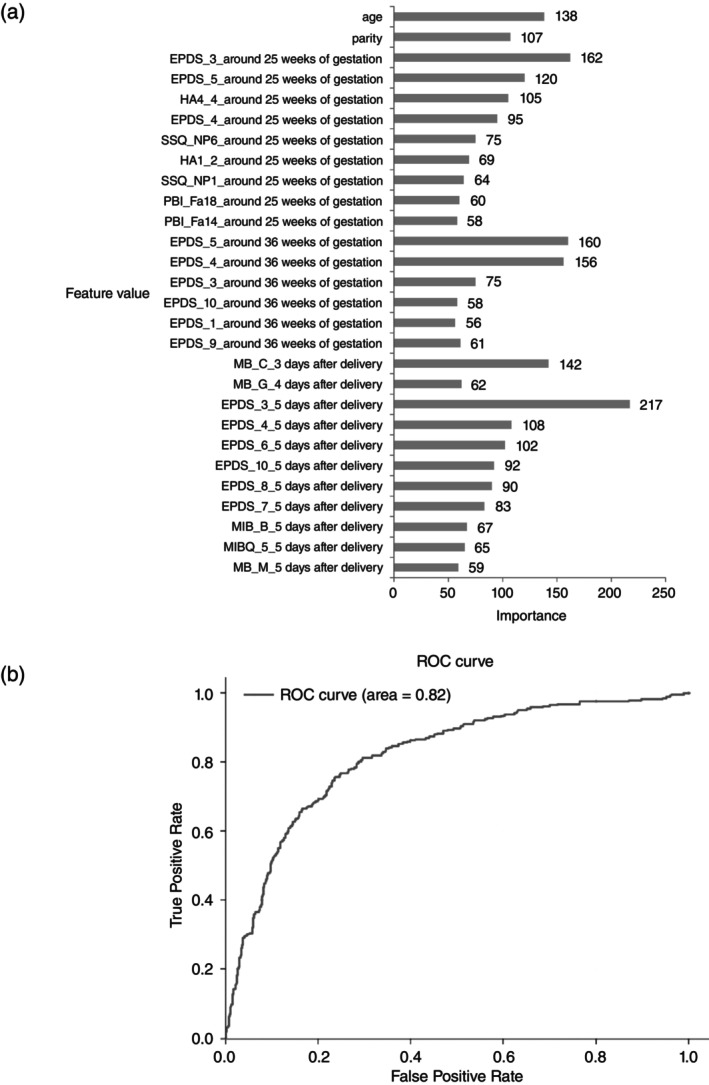
Ranked importance of predicters and AUC using the balanced GBDT (*n* = 1416). (a) Importance of predicters. (b) Receiver‐operating characteristic curve for predicting PPD based on the optimal predictive model developed using the balanced GBDT model. Area under the curve for PPD is 0.82. EPDS, Edinburgh postnatal depression scale; Fa, father; GBDT, gradient‐boosting decision tree; HA, Harm avoidance; MB, Stein's Scale; Mother‐Infant Bonding Questionnaire, MB_B,C,M,G, the item name of MB scale, number after the scale is each item number of the scales; NP, number of persons; PBI, parental bonding instrument; PPD, postpartum depression; SSQ, social support questionnaire.

In this study, we used machine learning technique and successfully constructed a highly accurate and convenient model for predicting depression in the first month after childbirth using data from pregnant women. In our model, we found that depression during pregnancy and immediately after delivery, maternity blues, age, parity, temperament, and other factors were important predictors. The prevalence of PPD is about 10%–15%, and the proportion of positives is smaller than the proportion of negatives. Therefore, the positive predictive value (PPV) tends to be low, as in previous studies^3^, ^4^ and this study. Future studies will need to develop a prediction tool with high PPV. In addition to the data used as predictors in this study, previous studies using machine learning reported that any history of depression is an important factor.^3^ In our previous studies, we also confirmed that a history of depression^5^ is a risk factor for PPD. However, since the number of responses to this item of our study was still small, we could not add it to this analysis. We think that it is important to try to build a model that includes this item to the predictor in the future.

We were able to build a simple and useful model for predicting PPD by using machine learning techniques. In the future, it will be important to build a more accurate model by adding data on the history of depression to the predictor.

## Disclosure statement

Norio Ozaki is an Editorial Board member of Psychiatry and Clinical Neurosciences and a co‐author of this article. To minimize bias, they were excluded from all editorial decision‐making related to the acceptance of this article for publication. Dr. Nakamura and Dr. Yamauchi have no conflict of interest. Dr. Ueno and Ichikawa has all support from SUSMED, Inc.; and Patents planned, issued or pending from SUSMED, Inc.; and stock or stock option of SUSMED, Inc. Dr. Takahashi has received a grant from Takeda and payments or honoraria for lectures from Jannsen Pharma, Sumitomo Pharma, Otsuka, Nobel Pharma, Takeda, Meiji Seika Pharma, Lundbeck Japan and Viatris. Dr. Ozaki has received grants from Sumitomo Pharma, Eisai, Otsuka, Shionogi, Mochida, KAITEKI, Takeda, Nihon Medi‐Physics, Eli Lilly Japan, Mitsubishi Tanabe, and DAIICHI SANKYO; and consulting fees from Sumitomo Pharma, Taisho Pharma, Boehringer Ingelheim, Otsuka, and Mochida; and payments or honoraria for lectures, presentations, speakers bureaus, manuscript writing or educational events from Sumitomo Pharma, Eisai, Otsuka, Mochida, Takeda, Meiji Seika Pharma, EA Pharma, MSD, Lundbeck Japan, Viatris, Kyowa Kirin, and TSUMURA.

## Supporting information


**Data S1.** Supporting information.

**Data S2.** Supporting information.

**Data S3.** Supporting information.
