# Supplementary material for: Early identification of postpartum depression using machine learning
Source: Psychiatry Clin Neurosci. 2024 Apr 15;78(6):372–3. doi: 10.1111/pcn.13659 (PMC11488638; doi:10.1111/pcn.13659)
Supplement: Supplementary file 1 — Data S1. Supporting information. [file PCN-78-372-s003.docx]

around 25 weeks of gestation

EPDS, MIBQ, TCI(HA), SSQ, PBI, age, parity, personal annual income, annual income of family

EPDS, MIBQ

around 36 weeks of gestation

MB

1 day after delivery

MB

2 days after delivery

3 days after delivery

MB

MB

4 days after delivery

EPDS, MIBQ, MB

5 days after delivery

EPDS

1 month after delivery

FigureS1. Flowchart of the study procedures

Abbreviations：EPDS, Edinburgh Postnatal Depression Scale, MIBQ, Mother-Infant Bonding Questionnaire, TCI, Temperament and Character Inventory, HA, Harm avoidance, SSQ, Social Support Questionnaire, PBI, Parental Bonding Instrument, MB, Stein’s Scale.

Women enrolled

n=1,559

Drop out around 25 weeks of gestation

n=1

Answering questionnaires

around 36 weeks of gestation

n=1,558

Drop out around 36 weeks of gestation

n=125

Answering questionnaires

5 days after delivery

n=1,433

Drop out 5 days after delivery

n=7

Answering questionnaires

1 month after delivery

n=1,426

There is a missing value in EPDS

one month after delivery

n=10

No missing values in EPDS

one month after delivery

n=1,416

not PPD

EPDS scored

8 points or less

n=1,122

PPD

EPDS scored

9 points or more

n=294

FigureS2. Flowchart of the recruitment process

Abbreviations：EPDS, Edinburgh Postnatal Depression Scale, PPD, postpartum depression.

Exact response rates are not possible to calculate because of the incomplete aggregation from August 2004 to March 2011. During the period from April 2011 to November 2020, we recruited 3,529 pregnant women and received consent from 988 women. The agreement rate for the entire research period is estimated to be around 30%.
