# Supplementary material for: Early identification of postpartum depression using machine learning
Source: Psychiatry Clin Neurosci. 2024 Apr 15;78(6):372–3. doi: 10.1111/pcn.13659 (PMC11488638; doi:10.1111/pcn.13659)
Supplement: Supplementary file 2 — Data S2. Supporting information. [file PCN-78-372-s001.docx]

Supplementary materials

**Early identification of postpartum depression using machine learning**

**Methods**

**Study Design**

The data in the present study were extracted from a prospective cohort study conducted in Nagoya, Japan from August 2004 to March 2020 that included a self-administered questionnaire survey. The questionnaire included the Edinburgh Postnatal Depression Scale (EPDS), Mother-Infant Bonding Questionnaire (MIBQ), Temperament and Character Inventory (TCI), Social Support Questionnaire (SSQ), The Parental Bonding Instrument (PBI), Stein’s Scale（MB）, and sociodemographic questions (age, parity, personal annual income and annual income of family).

The first questionnaire was completed in early pregnancy (around 25 weeks of gestation) and included the EPDS, MIBQ, TCI, SSQ, PBI and sociodemographic questions. In late pregnancy (around 36 weeks of gestation), EPDS and MIBQ were performed. 4 times in the 4 days after delivery (first, second, third and fourth), the MB was performed. 5 days after delivery, EPDS, MIBQ and MB were performed. MIBQ and MB were completed 5 days after delivery. At 1 month after delivery, the EPDS were completed. At 1 month after delivery, the completed questionnaire was returned by postal mail. The flowchart of the study procedures is shown in FigureS1. This study followed the STrengthening the Reporting of OBservational studies in Epidemiology (STROBE) reporting guideline.

**Participants**

Participants were recruited from one general hospital, two obstetrics and gynecology hospitals, and one university hospital. Pregnant women who were attending Perinatal and Childbirth Classes that started before the 25th week of pregnancy and who met the following eligibility criteria were asked to participate: 1) pregnant participants aged 20 years or older, and 2) ability to read and write Japanese. The age of adulthood in Japan is 20 years old. Therefore, in our study, we excluded pregnant women under the age of 20 because they are considered minors. Perinatal and Childbirth Classes are part of pregnancy care in Japan and are conducted mainly at gynecology hospitals and public health centers. Perinatal and Childbirth Classes are conducted by doctors, midwives, and other staff members to teach pregnant women about pregnancy, delivery, and childcare. Most pregnant women attend Perinatal and Childbirth Classes, but participation is voluntary.1,559 women participated in the study and 1,416 women responded to all 10 EPDS items one month after delivery. The flowchart of the recruitment process is shown in Figure S2.

**Measurements**

**EPDS**

The EPDS is a self-administered questionnaire designed by Cox et al. in 1987 to screen for PPD. EPDS comprises 10 items and is scored on a four-point Likert scale (from 0 to 3), with total scores ranging from 0 to 30.^1^ The reliability and construct validity of the Japanese version of the EPDS during the periods from pregnancy to postpartum were established^2^. In our cohort study, we used the Japanese version of the EPDS, developed by Okano et al. in 1996.^3^ scores of ≥9 were used to screen for minor and major depressive episodes (sensitivity: 75% and 82%, respectively; specificity: 93% and 95%, respectively).^3^ We evaluated depressive symptoms using the EPDS 1 month after birth.

**MIBQ**

Mother’s positive feeling towards their infant was called bonding. The MIBQ is a self-report questionnaire composed of nine items, with responses rated on a four-point Likert scale (from 0 to 3), with the scale for some items being reversed.^4, 5^ Total scores range from 0 to 27, with higher scores indicating poorer mother-to-infant bonding. The reliability and validity of the MIBQ in the pregnancy and postpartum periods has already been confirmed.

**TCI**

We used TCI to evaluate HA, which has been reported as being associated with depression.^6^ The TCI is a self-report questionnaire consisting of 125 items that look at four dimensions of temperament (novelty seeking, harm avoidance (HA), reward dependence, and persistence) and three dimensions of character (self-directedness, cooperativeness, and self-transcendence). We used the Japanese version of the TCI-125, which includes 125 questions covering 20 items pertaining to HA.^7^ Its reliability and validity have been previously confirmed.^8, 9^ HA scores ranged from 0 to 20.

**SSQ**

The Japanese version of the SSQ was used to measure perceived social support among mothers in the present study. It has confirmed reliability and validity for the pregnancy and postpartum periods^.10^ It has two factors, “Number of Persons(NP)” and “Satisfaction Rating(SR)”^11.^ NP and SR each consist of 6 items: NP measures the number of people who provide support, and SR measures satisfaction with support using a six-point Likert scale (1-6).

**PBI**

PBI is a self-report questionnaire that evaluates the parent’s perceptions of how they were raised by recalling how they were treated before 16 years of age. The PBI consists of 25 items, with two factors（Care and Overprotection） derived from factor analyses.^12^ The Japanese version was used in this study. Its reliability and validity has been confirmed.^13^

**MB**

Stein’s Scale was used to assess Maternity Blues(MB).^14^ This scale has 13 items, with a total score ranging from 0 to 26. The Japanese version was used in this study. It has proven validity and reliability for detecting MB in the early postpartum period.^15^ The cut-off point was 7/8, with scores of 8 or higher indicating mood swings or MB.^14^ Since MB is transitory in nature, it is recommended that self-evaluation be conducted on consecutive days after delivery.^14, 16^ In this study, mothers completed Stein’s Scale on each of the first 5 days after delivery.

**Missing Data Imputation**

The missing percentages for each item are shown in Table S3. Stata command *mcartest* was used for testing missing at random assumption for total scores of each measurements, which showed missing values were at random (Chi-square distance = 1050.1400, Degrees of freedom = 1031, P = 0.3323). For each imputed variable the multiple imputations are carried out using a regression model (fully conditional specification).^17^ Missing values were filled in using a regression model in which variables other than those to be complemented were used as variables.^18^ Logistic regression analysis was used for categorical variables, and multiple regression analysis was used for continuous value variables.

**Machine Learning**

We used EPDS, MIBQ, TCI, SSQ, PBI, MB, and sociodemographic questions (age, parity, personal annual income, and annual income of the family) as feature values in our predictive model. We defined PPD as a subject with a total EPDS score of 9 or higher at 1 month after delivery, and not PPD as a subject with a total EPDS score of 8 or lower at 1 month after delivery. To estimate the importance of variables for the predictive modeling, we calculated variable importance. The variable importance was assessed using the mean decrease in the gain.

We used the L1 penalized logistic regression model^19^ to build the baseline model. While logistic regression is a well-known method, it has a shortcoming in that it may lead to further degradation when there is a high correlation between features. L1 penalized logistic regression overcomes the disadvantage and increases the model stability.^20^ Our model also included features extracted by the RuleFit algorithm. The RuleFit algorithm learns sparse linear models that include automatically detected interaction effects in the form of decision rules^21^. RuleFit is composed of two steps: 1. Extracting decision rules from original features for the purpose, random forest was used in the present study^22^. 2. Build predictive models using sparse linear models with the original features and new features corresponding to the decision rules. In the study, we used the L1 penalized logistic regression model as a sparse linear model^23^. The reason for using the L1 regression model here makes sense for feature selection. These new features are reflected in the interactions between the original features.

In addition to the logistic regression model, we used decision tree, gradient-boosting decision tree (GBDT) and balanced GBDT as machine learning methods. Ensemble learning refers to an algorithm that combines base learners (such as decision trees and linear classifiers): the GBDT approaches are examples of such learning.^24^ We adopted the GBDT approach because of its superior predictive ability.^25^ GBDT yields a predictive model in the form of an ensemble of decision trees and affords strong predictive power with a differentiable loss function. Use of the GBDT for predictions required that we tune the parameters, such as the maximum depth of trees. The parameters were determined by a Bayesian optimization.

PPD is generally considered to be observable in approximately 10% of mothers,^26, 27^ and it was expected that the data set of the present study would be dominated by the non-PPD group relative to PPD group. In general, predictive models trained on such unbalanced data sets often produce biased results. It is known that the best predictive models in unbalanced data are those that combine undersampling with ensemble learning. Therefore, in this study, we used GBDT for training datasets with equal numbers of PPD and non-PPD using undersampling (balanced GBDT).

Optuna, a Python library, was used for hyperparameter tuning by Bayesian optimization, scikit-learn,^21^ a Python library, was used for L1 regularized logistic regression, GBDT, RuleFit, and decision trees, and Python 3.7.3, was used for other data handling.

**Evaluation metrics**

We used an eight-fold external cross-validation procedure and averaged the results to assess the predictive ability of each machine learning algorithm. For the cross-validation procedure, we used subject-wise, rather than record-wise, data splitting, since identity confounding was reported.^22, 23^ We then used a receiver-operating characteristics (ROC) curve analysis to assess the predictive accuracy of our classifier. The area under the curve (AUC) was evaluated as the ability to predict PPD.^28, 29^Additionally, we calculated accuracy, sensitivity, specificity, positive predictive value (PPV), and negative predictive value (NPV) to evaluate our predictive model.

**Ethical Consideration**

A verbal and written explanation of the study was given to all participants, and written informed consent was obtained from all those who agreed to participate. The study protocol was approved by the Ethics Committee of the Nagoya University Graduate School of Medicine. All study methods met the Committee’s guidelines and regulations.

**Limitation**

This study has some limitations. First, selection bias was possible because the target facilities were limited to four hospitals. In addition, we could not include pregnant women who did not participate in Perinatal and Childbirth Classes. Second, the psychiatric variables were evaluated based on a self-report questionnaire, not on a diagnosis by a psychiatrist. Third, pregnancy-related complications^30^ have been identified as risk factors for PPD, but were not included in this analysis. We are currently analyzing these data with an obstetrician, and hope to include them in a future issue. Fourth, our results were obtained from Japanese pregnant women. It is debatable whether these findings can be generalized.

**References**

1. Cox JL, Holden JM, Sagovsky R. Detection of postnatal depression. Development of the 10-item Edinburgh Postnatal Depression Scale. *Br J Psychiatry* 1987; **150**: 782-6.

2. Kubota C, Inada T, Nakamura Y et al. Stable factor structure of the Edinburgh Postnatal Depression Scale during the whole peripartum period: Results from a Japanese prospective cohort study. *Sci Rep* 2018; **8**: 17659.

3. Okano T, Murata M, Masuji F et al. Validation and reliability of Japanese version of the EPDS. *Arch Psychiatr Diag Clin Eval* 1996; **7**: 523-533.

4. Taylor A, Atkins R, Kumar R, Adams D, Glover V. A new Mother-to-Infant Bonding Scale: links with early maternal mood. *Arch Womens Ment Health* 2005; **8**: 45-51.

5. Ohara M, Okada T, Kubota C et al. Validation and factor analysis of mother-infant bonding questionnaire in pregnant and postpartum women in Japan. *BMC Psychiatry* 2016; **16**: 212.

6. Furumura K, Koide T, Okada T et al. Prospective study on the association between harm avoidance and postpartum depressive state in a maternal cohort of Japanese women. *PloS one* 2012; **7**: e34725.

7. Kijima N, Saito R, Takeuchi M et al. Cloninger’s seven-factor model of temperament and character and Jananese version of Temperament and Character Inventory (TCI). *Arch Psychiatr Diagn Clin Eval* 1996; **7**: 379-399.

8. Kijima N, Tanaka E, Suzuki N, Higuchi H, Kitamura T. Reliability and validity of the Japanese version of the Temperament and Character Inventory. *Psychol Rep* 2000; **86**: 1050-8.

9. Takeuchi M, Miyaoka H, Tomoda A, Suzuki M, Lu X, Kitamura T. Validity and reliability of the Japanese version of the Temperament and Character Inventory: a study of university and college students. *Compr Psychiatry* 2011; **52**: 109-17.

10. Furukawa TA, Harai H, Hirai T, Kitamura T, Takahashi K. Social Support Questionnaire among psychiatric patients with various diagnoses and normal controls. *Soc Psychiatry Psychiatr Epidemiol* 1999; **34**: 216-22.

11. Morikawa M, Okada T, Ando M et al. Relationship between social support during pregnancy and postpartum depressive state: a prospective cohort study. *Sci Rep* 2015; **5**: 10520.

12. Parker G. Parental characteristics in relation to depressive disorders. *Br J Psychiatry* 1979; **134**: 138-47.

13. Kitamura T, Suzuki T. A validation study of the Parental Bonding Instrument in a Japanese population. *Jpn J Psychiatry Neurol* 1993; **47**: 29-36.

14. Stein GS. The pattern of mental change and body weight change in the first post-partum week. *J Psychosom Res* 1980; **24**: 165-71.

15. Okano T, Nomura J, Koshikawa N, Doi M, Tatsunuma T. Cross-cultural study of materni- ty blues and postpartum depression (in Japa- nese). *Clin Psychiatry* 1991; **33**: 1051-1058.

16. Kennerley H, Gath D. Maternity blues. I. Detection and measurement by questionnaire. *Br J Psychiatry* 1989; **155**: 356-62.

17. van Buuren S, Groothuis-Oudshoorn K. mice: Multivariate Imputation by Chained Equations in R. *Journal of Statistical Software* 2011; **45**: 1 - 67.

18. van der Heijden GJMG, Donders ART, Stijnen T, Moons KGM. Imputation of missing values is superior to complete case analysis and the missing-indicator method in multivariable diagnostic research: A clinical example. *J Clin Epidemiol* 2006; **59**: 1102-1109.

19. Lecessie S, Vanhouwelingen JC. Ridge Estimators in Logistic-Regression. *Appl Stat-J Roy St C* 1992; **41**: 191-201.

20. Liu Z, Shen YY, Ott J. Multilocus association mapping using generalized ridge logistic regression. *Bmc Bioinformatics* 2011; **12**.

21. Fabian P, Gael V, Alexandre G, Vincent M, Bertrand T. Scikit-learn: Machine Learning in Python. *Journal of Machine Learning Research* 2011; **12**: 2825-2830.

22. Saeb S, Lonini L, Jayaraman A, Mohr DC, Kording KP. The need to approximate the use-case in clinical machine learning. *Gigascience* 2017; **6**: 1-9.

23. Neto EC, Pratap A, Perumal TM et al. Detecting the impact of subject characteristics on machine learning-based diagnostic applications. *Npj Digit Med* 2019; **2**.

24. Friedman JH. Stochastic gradient boosting. *Comput Stat Data An* 2002; **38**: 367-378.

25. Natekin A, Knoll A. Gradient boosting machines, a tutorial. *Front Neurorobotics* 2013; **7**.

26. O'Hara MW, Swain AM. Rates and risk of postpartum depression--a meta-analysis. *International Review of Psychiatry* 1996; **8**: 37.

27. Ishikawa N, Goto S, Murase S et al. Prospective study of maternal depressive symptomatology among Japanese women. *J Psychosom Res* 2011; **71**: 264-9.

28. Swets JA. Measuring the accuracy of diagnostic systems. *Science* 1988; **240**: 1285-93.

29. Fischer JE, Bachmann LM, Jaeschke R. A readers' guide to the interpretation of diagnostic test properties: clinical example of sepsis. *Intens Care Med* 2003; **29**: 1043-1051.

30. Boyce PM, Todd AL. Increased risk of postnatal depression after emergency caesarean section. *Med J Aust* 1992; **157**: 172-4.
