# Supplementary material for: Early identification of postpartum depression using machine learning
Source: Psychiatry Clin Neurosci. 2024 Apr 15;78(6):372–3. doi: 10.1111/pcn.13659 (PMC11488638; doi:10.1111/pcn.13659)
Supplement: Supplementary file 3 — Data S3. Supporting information. [file PCN-78-372-s002.docx]

Table S1. Comparison of predictors of PPD and not PPD

†: chi-square test between PPD and not PPD. ‡: Student’s *t*-test between PPD and not PPD.

|  | PPD (N=294) | | not PPD(N=1,122) | | chi-square test |
| --- | --- | --- | --- | --- | --- |
|  | n | % | n | % | *p*-value † |
| parity |  |  |  |  |  |
| primiparas | 238 | 83.2 | 794 | 72.6 | < .001 |
| multiparas | 48 | 16.8 | 300 | 27.4 |  |
|  | PPD (N=294) | | not PPD(N=1,122) | | Student’s *t*-test |
|  | n | Mean ±SD | n | Mean ±SD | *p*-value ‡ |
| Age (years) | 293 | 31.9 ± 4.4 | 1107 | 32.6 ± 4.6 | 0.02 |
| EPDS total score around 25 weeks of gestation | 291 | 8.3 ± 5.7 | 1100 | 4.0 ± 3.8 | < .001 |
| EPDS total score around 36 weeks of gestation | 287 | 7.5 ± 5.3 | 1088 | 3.4 ± 3.5 | < .001 |
| EPDS total score at 5 days after delivery | 289 | 9.4 ± 5.7 | 1101 | 3.9 ± 4.0 | < .001 |
| HA(TCI) score around 25 weeks of gestation | 290 | 13.3 ± 4.6 | 1093 | 11.0 ± 4.4 | < .001 |
| SSQ NP total score around 25 weeks of gestation | 291 | 21.5 ± 14.5 | 1107 | 24.1 ± 13.2 | < .001 |
| SSQ SR total score around 25 weeks of gestation | 285 | 28.5 ± 7.2 | 1092 | 29.9 ± 6.8 | < .001 |
| PBI Fa total score around 25 weeks of gestation | 284 | 34.1 ± 8.7 | 1076 | 34.8 ± 7.3 | 0.242 |
| PBI Mo total score around 25 weeks of gestation | 287 | 39.4 ± 8.0 | 1096 | 39.7 ± 6.4 | 0.523 |
| MIBQ total score around 25 weeks of gestation | 290 | 4.0 ± 3.7 | 1086 | 3.1 ± 3.0 | < .001 |
| MIBQ total score around 36 weeks of gestation | 285 | 3.8 ± 3.5 | 1074 | 3.0 ± 2.9 | < .001 |
| MIBQ total score at 5 days after delivery | 286 | 2.4 ± 2.8 | 1089 | 1.5 ± 1.9 | < .001 |
| MB total score at 1 day after delivery | 291 | 5.8 ± 4.3 | 1083 | 3.6 ± 3.5 | < .001 |
| MB total score at 2 days after delivery | 290 | 6.3 ± 4.6 | 1086 | 3.5 ± 3.5 | < .001 |
| MB total score at 3 days after delivery | 289 | 6.8 ± 4.8 | 1090 | 3.4 ± 3.4 | < .001 |
| MB total score at 4 days after delivery | 286 | 6.6 ± 4.6 | 1090 | 3.4 ± 3.4 | < .001 |
| MB total score at 5 days after delivery | 285 | 6.9 ± 5.0 | 1098 | 3.3 ± 3.3 | < .001 |

Abbreviations：PPD, postpartum depression, EPDS, Edinburgh Postnatal Depression Scale, MIBQ, Mother-Infant Bonding Questionnaire, TCI, Temperament and Character Inventory, HA, Harm avoidance, SSQ, Social Support Questionnaire, NP, Number of Persons, SR, Satisfaction Rating, PBI, Parental Bonding Instrument, Fa, father, Mo, mother, MB, Stein’s Scale.

Table S2. Various indicators of Predictive Models

| model | accuracy | AUC | AUC_95% confidence interval | | sensitivity | specificity | PPV | NPV |
| --- | --- | --- | --- | --- | --- | --- | --- | --- |
|  |  |  | lower limit | upper limit |  |  |  |  |
| logistic regression | 71.69% | 0.8285 | 0.803 | 0.854 | 80.0% | 69.5% | 40.7% | 93.0% |
| decision tree | 60.56% | 0.7291 | 0.697 | 0.761 | 80.0% | 55.5% | 32.0% | 91.4% |
| GBDT | 72.90% | 0.8132 | 0.786 | 0.840 | 80.0% | 71.0% | 42.0% | 93.1% |
| balanced GBDT | 73.11% | 0.8195 | 0.792 | 0.847 | 80.0% | 71.3% | 42.2% | 93.2% |

Abbreviations：AUC, area under the curve; PPV, positive predictive value; NPV, negative predictive value; GBDT, gradient-boosting decision tree

Table S3. The missing percentages for each item

| predictor | Number of missing values | missing percentage |
| --- | --- | --- |
| age | 16 | 1.13 |
| parity | 36 | 2.54 |
| annual personal income | 165 | 11.65 |
| annual household income | 160 | 11.30 |
| around 25 weeks of gestation_HA1_1 | 22 | 1.55 |
| around 25 weeks of gestation_HA2_1 | 22 | 1.55 |
| around 25 weeks of gestation_HA4_1 | 25 | 1.77 |
| around 25 weeks of gestation_HA3_1 | 22 | 1.55 |
| around 25 weeks of gestation_HA3_2 | 22 | 1.55 |
| around 25 weeks of gestation_HA2_2 | 23 | 1.62 |
| around 25 weeks of gestation_HA3_3 | 22 | 1.55 |
| around 25 weeks of gestation_HA1_2 | 22 | 1.55 |
| around 25 weeks of gestation_HA1_3 | 22 | 1.55 |
| around 25 weeks of gestation_HA4_2 | 23 | 1.62 |
| around 25 weeks of gestation_HA1_4 | 22 | 1.55 |
| around 25 weeks of gestation_HA2_3 | 22 | 1.55 |
| around 25 weeks of gestation_HA3_4 | 25 | 1.77 |
| around 25 weeks of gestation_HA4_3 | 26 | 1.84 |
| around 25 weeks of gestation_HA1_5 | 22 | 1.55 |
| around 25 weeks of gestation_HA3_5 | 22 | 1.55 |
| around 25 weeks of gestation_HA4_4 | 22 | 1.55 |
| around 25 weeks of gestation_HA2_4 | 22 | 1.55 |
| around 25 weeks of gestation_HA2_5 | 22 | 1.55 |
| around 25 weeks of gestation_HA4_5 | 22 | 1.55 |
| around 25 weeks of gestation_SSQ_NP1 | 16 | 1.13 |
| around 25 weeks of gestation_SSQ_SR1 | 27 | 1.91 |
| around 25 weeks of gestation_SSQ_NP2 | 16 | 1.13 |
| around 25 weeks of gestation_SSQ_SR2 | 27 | 1.91 |
| around 25 weeks of gestation_SSQ_NP3 | 16 | 1.13 |
| around 25 weeks of gestation_SSQ_SR3 | 25 | 1.77 |
| around 25 weeks of gestation_SSQ_NP4 | 16 | 1.13 |
| around 25 weeks of gestation_SSQ_SR4 | 23 | 1.62 |
| around 25 weeks of gestation_SSQ_NP5 | 18 | 1.27 |
| around 25 weeks of gestation_SSQ_SR5 | 26 | 1.84 |
| around 25 weeks of gestation_SSQ_NP6 | 16 | 1.13 |
| around 25 weeks of gestation_SSQ_SR6 | 27 | 1.91 |
| around 25 weeks of gestation_PBI_Fa1 | 27 | 1.91 |
| around 25 weeks of gestation_PBI_Fa2 | 29 | 2.05 |
| around 25 weeks of gestation_PBI_Fa3 | 29 | 2.05 |
| around 25 weeks of gestation_PBI_Fa4 | 32 | 2.26 |
| around 25 weeks of gestation_PBI_Fa5 | 33 | 2.33 |
| around 25 weeks of gestation_PBI_Fa6 | 29 | 2.05 |
| around 25 weeks of gestation_PBI_Fa7 | 32 | 2.26 |
| around 25 weeks of gestation_PBI_Fa8 | 34 | 2.40 |
| around 25 weeks of gestation_PBI_Fa9 | 29 | 2.05 |
| around 25 weeks of gestation_PBI_Fa10 | 29 | 2.05 |
| around 25 weeks of gestation_PBI_Fa11 | 30 | 2.12 |
| around 25 weeks of gestation_PBI_Fa12 | 30 | 2.12 |
| around 25 weeks of gestation_PBI_Fa13 | 29 | 2.05 |
| around 25 weeks of gestation_PBI_Fa14 | 31 | 2.19 |
| around 25 weeks of gestation_PBI_Fa15 | 33 | 2.33 |
| around 25 weeks of gestation_PBI_Fa16 | 29 | 2.05 |
| around 25 weeks of gestation_PBI_Fa17 | 31 | 2.19 |
| around 25 weeks of gestation_PBI_Fa18 | 30 | 2.12 |
| around 25 weeks of gestation_PBI_Fa19 | 34 | 2.40 |
| around 25 weeks of gestation_PBI_Fa20 | 29 | 2.05 |
| around 25 weeks of gestation_PBI_Fa21 | 29 | 2.05 |
| around 25 weeks of gestation_PBI_Fa22 | 31 | 2.19 |
| around 25 weeks of gestation_PBI_Fa23 | 28 | 1.98 |
| around 25 weeks of gestation_PBI_Fa24 | 29 | 2.05 |
| around 25 weeks of gestation_PBI_Fa25 | 29 | 2.05 |
| around 25 weeks of gestation_PBI_Mo1 | 13 | 0.92 |
| around 25 weeks of gestation_PBI_Mo2 | 14 | 0.99 |
| around 25 weeks of gestation_PBI_Mo3 | 15 | 1.06 |
| around 25 weeks of gestation_PBI_Mo4 | 15 | 1.06 |
| around 25 weeks of gestation_PBI_Mo5 | 14 | 0.99 |
| around 25 weeks of gestation_PBI_Mo6 | 14 | 0.99 |
| around 25 weeks of gestation_PBI_Mo7 | 15 | 1.06 |
| around 25 weeks of gestation_PBI_Mo8 | 14 | 0.99 |
| around 25 weeks of gestation_PBI_Mo9 | 13 | 0.92 |
| around 25 weeks of gestation_PBI_Mo10 | 13 | 0.92 |
| around 25 weeks of gestation_PBI_Mo11 | 13 | 0.92 |
| around 25 weeks of gestation_PBI_Mo12 | 15 | 1.06 |
| around 25 weeks of gestation_PBI_Mo13 | 14 | 0.99 |
| around 25 weeks of gestation_PBI_Mo14 | 15 | 1.06 |
| around 25 weeks of gestation_PBI_Mo15 | 15 | 1.06 |
| around 25 weeks of gestation_PBI_Mo16 | 13 | 0.92 |
| around 25 weeks of gestation_PBI_Mo17 | 16 | 1.13 |
| around 25 weeks of gestation_PBI_Mo18 | 13 | 0.92 |
| around 25 weeks of gestation_PBI_Mo19 | 15 | 1.06 |
| around 25 weeks of gestation_PBI_Mo20 | 14 | 0.99 |
| around 25 weeks of gestation_PBI_Mo21 | 14 | 0.99 |
| around 25 weeks of gestation_PBI_Mo22 | 13 | 0.92 |
| around 25 weeks of gestation_PBI_Mo23 | 13 | 0.92 |
| around 25 weeks of gestation_PBI_Mo24 | 13 | 0.92 |
| around 25 weeks of gestation_PBI_Mo25 | 13 | 0.92 |
| around 25 weeks of gestation_EPDS1 | 16 | 1.13 |
| around 25 weeks of gestation_EPDS2 | 16 | 1.13 |
| around 25 weeks of gestation_EPDS3 | 15 | 1.06 |
| around 25 weeks of gestation_EPDS4 | 16 | 1.13 |
| around 25 weeks of gestation_EPDS5 | 17 | 1.20 |
| around 25 weeks of gestation_EPDS6 | 17 | 1.20 |
| around 25 weeks of gestation_EPDS7 | 16 | 1.13 |
| around 25 weeks of gestation_EPDS8 | 19 | 1.34 |
| around 25 weeks of gestation_EPDS9 | 15 | 1.06 |
| around 25 weeks of gestation_EPDS10 | 15 | 1.06 |
| around 25 weeks of gestation_MIBQ1 | 15 | 1.06 |
| around 25 weeks of gestation_MIBQ2 | 15 | 1.06 |
| around 25 weeks of gestation_MIBQ3 | 24 | 1.69 |
| around 25 weeks of gestation_MIBQ4 | 17 | 1.20 |
| around 25 weeks of gestation_MIBQ5 | 16 | 1.13 |
| around 25 weeks of gestation_MIBQ6 | 22 | 1.55 |
| around 25 weeks of gestation_MIBQ7 | 15 | 1.06 |
| around 25 weeks of gestation_MIBQ8 | 21 | 1.48 |
| around 25 weeks of gestation_MIBQ9 | 20 | 1.41 |
| around 36 weeks of gestation_EPDS1 | 31 | 2.19 |
| around 36 weeks of gestation_EPDS2 | 33 | 2.33 |
| around 36 weeks of gestation_EPDS3 | 31 | 2.19 |
| around 36 weeks of gestation_EPDS4 | 32 | 2.26 |
| around 36 weeks of gestation_EPDS5 | 31 | 2.19 |
| around 36 weeks of gestation_EPDS6 | 33 | 2.33 |
| around 36 weeks of gestation_EPDS7 | 33 | 2.33 |
| around 36 weeks of gestation_EPDS8 | 34 | 2.40 |
| around 36 weeks of gestation_EPDS9 | 32 | 2.26 |
| around 36 weeks of gestation_EPDS10 | 32 | 2.26 |
| around 36 weeks of gestation_MIBQ1 | 34 | 2.40 |
| around 36 weeks of gestation_MIBQ2 | 32 | 2.26 |
| around 36 weeks of gestation_MIBQ3 | 37 | 2.61 |
| around 36 weeks of gestation_MIBQ4 | 35 | 2.47 |
| around 36 weeks of gestation_MIBQ5 | 32 | 2.26 |
| around 36 weeks of gestation_MIBQ6 | 40 | 2.82 |
| around 36 weeks of gestation_MIBQ7 | 33 | 2.33 |
| around 36 weeks of gestation_MIBQ8 | 32 | 2.26 |
| around 36 weeks of gestation_MIBQ9 | 32 | 2.26 |
| 1 day after delivery_MBA | 28 | 1.98 |
| 1 day after delivery_MBB | 29 | 2.05 |
| 1 day after delivery_MBC | 31 | 2.19 |
| 1 day after delivery_MBD | 28 | 1.98 |
| 1 day after delivery_MBE | 29 | 2.05 |
| 1 day after delivery_MBF | 29 | 2.05 |
| 1 day after delivery_MBG | 33 | 2.33 |
| 1 day after delivery_MBH | 29 | 2.05 |
| 1 day after delivery_MBI | 27 | 1.91 |
| 1 day after delivery_MBJ | 27 | 1.91 |
| 1 day after delivery_MBK | 27 | 1.91 |
| 1 day after delivery_MBL | 27 | 1.91 |
| 1 day after delivery_MBM | 28 | 1.98 |
| 2 days after delivery_MBA | 27 | 1.91 |
| 2 days after delivery_MBB | 27 | 1.91 |
| 2 days after delivery_MBC | 25 | 1.77 |
| 2 days after delivery_MBD | 25 | 1.77 |
| 2 days after delivery_MBE | 28 | 1.98 |
| 2 days after delivery_MBF | 26 | 1.84 |
| 2 days after delivery_MBG | 32 | 2.26 |
| 2 days after delivery_MBH | 27 | 1.91 |
| 2 days after delivery_MBI | 25 | 1.77 |
| 2 days after delivery_MBJ | 25 | 1.77 |
| 2 days after delivery_MBK | 26 | 1.84 |
| 2 days after delivery_MBL | 25 | 1.77 |
| 2 days after delivery_MBM | 26 | 1.84 |
| 3 days after delivery_MBA | 24 | 1.69 |
| 3 days after delivery_MBB | 24 | 1.69 |
| 3 days after delivery_MBC | 24 | 1.69 |
| 3 days after delivery_MBD | 25 | 1.77 |
| 3 days after delivery_MBE | 27 | 1.91 |
| 3 days after delivery_MBF | 24 | 1.69 |
| 3 days after delivery_MBG | 28 | 1.98 |
| 3 days after delivery_MBH | 25 | 1.77 |
| 3 days after delivery_MBI | 23 | 1.62 |
| 3 days after delivery_MBJ | 23 | 1.62 |
| 3 days after delivery_MBK | 23 | 1.62 |
| 3 days after delivery_MBL | 23 | 1.62 |
| 3 days after delivery_MBM | 24 | 1.69 |
| 4 days after delivery_MBA | 21 | 1.48 |
| 4 days after delivery_MBB | 20 | 1.41 |
| 4 days after delivery_MBC | 20 | 1.41 |
| 4 days after delivery_MBD | 22 | 1.55 |
| 4 days after delivery_MBE | 27 | 1.91 |
| 4 days after delivery_MBF | 21 | 1.48 |
| 4 days after delivery_MBG | 26 | 1.84 |
| 4 days after delivery_MBH | 24 | 1.69 |
| 4 days after delivery_MBI | 22 | 1.55 |
| 4 days after delivery_MBJ | 22 | 1.55 |
| 4 days after delivery_MBK | 22 | 1.55 |
| 4 days after delivery_MBL | 23 | 1.62 |
| 4 days after delivery_MBM | 24 | 1.69 |
| 5 days after delivery_MBA | 20 | 1.41 |
| 5 days after delivery_MBB | 22 | 1.55 |
| 5 days after delivery_MBC | 19 | 1.34 |
| 5 days after delivery_MBD | 20 | 1.41 |
| 5 days after delivery_MBE | 21 | 1.48 |
| 5 days after delivery_MBF | 20 | 1.41 |
| 5 days after delivery_MBG | 25 | 1.77 |
| 5 days after delivery_MBH | 23 | 1.62 |
| 5 days after delivery_MBI | 20 | 1.41 |
| 5 days after delivery_MBJ | 21 | 1.48 |
| 5 days after delivery_MBK | 21 | 1.48 |
| 5 days after delivery_MBL | 20 | 1.41 |
| 5 days after delivery_MBM | 21 | 1.48 |
| 5 days after delivery_EPDS1 | 19 | 1.34 |
| 5 days after delivery_EPDS2 | 19 | 1.34 |
| 5 days after delivery_EPDS3 | 19 | 1.34 |
| 5 days after delivery_EPDS4 | 19 | 1.34 |
| 5 days after delivery_EPDS5 | 23 | 1.62 |
| 5 days after delivery_EPDS6 | 25 | 1.77 |
| 5 days after delivery_EPDS7 | 24 | 1.69 |
| 5 days after delivery_EPDS8 | 23 | 1.62 |
| 5 days after delivery_EPDS9 | 23 | 1.62 |
| 5 days after delivery_EPDS10 | 23 | 1.62 |
| 5 days after delivery_MIBQ1 | 21 | 1.48 |
| 5 days after delivery_MIBQ2 | 20 | 1.41 |
| 5 days after delivery_MIBQ3 | 25 | 1.77 |
| 5 days after delivery_MIBQ4 | 24 | 1.69 |
| 5 days after delivery_MIBQ5 | 20 | 1.41 |
| 5 days after delivery_MIBQ6 | 28 | 1.98 |
| 5 days after delivery_MIBQ7 | 21 | 1.48 |
| 5 days after delivery_MIBQ8 | 25 | 1.77 |
| 5 days after delivery_MIBQ9 | 21 | 1.48 |

Abbreviations：HA, Harm avoidance; SSQ, Social Support Questionnaire; NP, Number of Persons; SR, Satisfaction Rating; PBI, Parental Bonding Instrument; Fa, father; Mo, mother; EPDS, Edinburgh Postnatal Depression Scale; MIBQ, Mother-Infant Bonding Questionnaire; number after the scale is each item number of the scales. MB A,B,C,D,E,F,G,H,I,J,K,L,M the item name of MB scale,
